# Risk factors of postoperative complications after F/B-TEVAR for aortic arch lesions: a multicenter retrospective analysis

**DOI:** 10.3389/fcvm.2026.1737695

**Published:** 2026-04-02

**Authors:** Jianhang Hu, Yuexue Han, Yuzhu Wang, Yi Jin, Zihe Zhao, Zhao Liu

**Affiliations:** Department of Vascular Surgery, Nanjing University Medical School Affiliated Nanjing Drum Tower Hospital, Nanjing, China

**Keywords:** 3D-printing, aortic arch, complication, F/B-TEVAR, PMSG, risk factors

## Abstract

**Background:**

Fenestrated/Branched Thoracic Endovascular Aortic Repair (F/B-TEVAR) has emerged as a cornerstone treatment for various acute and subacute complex aortic arch diseases, but the high complication rate of F/B-TEVAR surgery continues to plague many patients.

**Methods:**

A retrospective analysis was conducted on 246 patients with aortic arch lesions who underwent F/B-TEVAR across 4 hospitals from February 2018 to December 2024. Case information and follow-up outcomes were analyzed using multivariate correlation calculation and multivariate logistic regression to summarize the risk factors associated with postoperative complications.

**Results:**

Postoperative complications were observed in 53 cases (21.5%). Statistically significant risk factors (all *P* < 0.05) included history of previous cerebral infarction, history of smoking, lesions involving branch arteries, and the aortic wall pathology (aortic atherosclerotic plaque, minute intermural hematomas, mural thrombus).

**Conclusions:**

This study identifies key independent risk factors for complications post F/B-TEVAR, emphasizing the potential benefit of 3D printing-assisted technology in reducing these risks. Further research is needed to validate these findings, which can help clinicians identify high-risk patients and implement appropriate interventions.

## 1 Introduction

Fenestrated/Branched Thoracic Endovascular Aortic Repair (F/B-TEVAR) has emerged as a cornerstone treatment for various acute and subacute complex aortic arch diseases, encompassing conditions such as aortic dissection, true aortic aneurysms, branch aneurysms, and other thoracic aortic disorders requiring proximal sealing and reconstruction of vital branch arteries in zone 2 or more proximal. Compared to open surgery, F/B-TEVAR offers advantages including reduced trauma, quicker recovery, and suitability for critically ill patients. However, TEVAR carries a notable complication rate, estimated as high as 38%, with common issues including endoleak, retrograde aortic tears, cerebrovascular events, spinal cord ischemia, and post-implantation syndrome (1). While extensive studies on F/B-TEVAR have been conducted, a definitive analysis of associated risks remains elusive. We retrospectively reviewed 246 cases treated between February 2018 and December 2024 across 4 hospitals using physician-modified stent-grafts (PMSG) and F/B-TEVAR for thoracic aortic arch lesions. Our analysis included the frequency of complications and a discussion of associated risk factors, detailed in the following section.

## 2 Methods

### 2.1 General information

From February 2018 to December 2024, 246 patients underwent F/B-TEVAR for thoracic aortic lesions at 4 medical centers in China. Clinical data were collected from each center, with aortic imaging and laboratory data obtained from respective departments. Intraoperative modification of PMSG was performed for all cases. The collection of postoperative case information was conducted in accordance with approvals from the medical ethics committees of each center, with all patients providing informed consent before surgery. Details of underlying diseases and comorbidities are presented in Table 2.

### 2.2 Patient inclusion criteria

- Patients diagnosed preoperatively with thoracic aortic dissection, thoracic aortic aneurysm, thoracic aortic ulcer, postoperative endoleak of thoracic aorta, intermural hematoma of thoracic aorta, or other thoracic aortic pathologies requiring surgical intervention;
- They must have undergone F/B-TEVAR;
- Those who demonstrated acceptable results in postoperative follow-up;
- Those who had provided relevant informed consent.

### 2.3 Patient exclusion criteria

- Acute thoracic aortic disease due to trauma;
- Those with malignant tumors and a life expectancy of less than 2 years;
- Those who diagnosed with Marfan's syndrome;
- Those with infected thoracic aortic aneurysm or who were pregnant.

### 2.4 Standardized procedure

**Figure 1:**
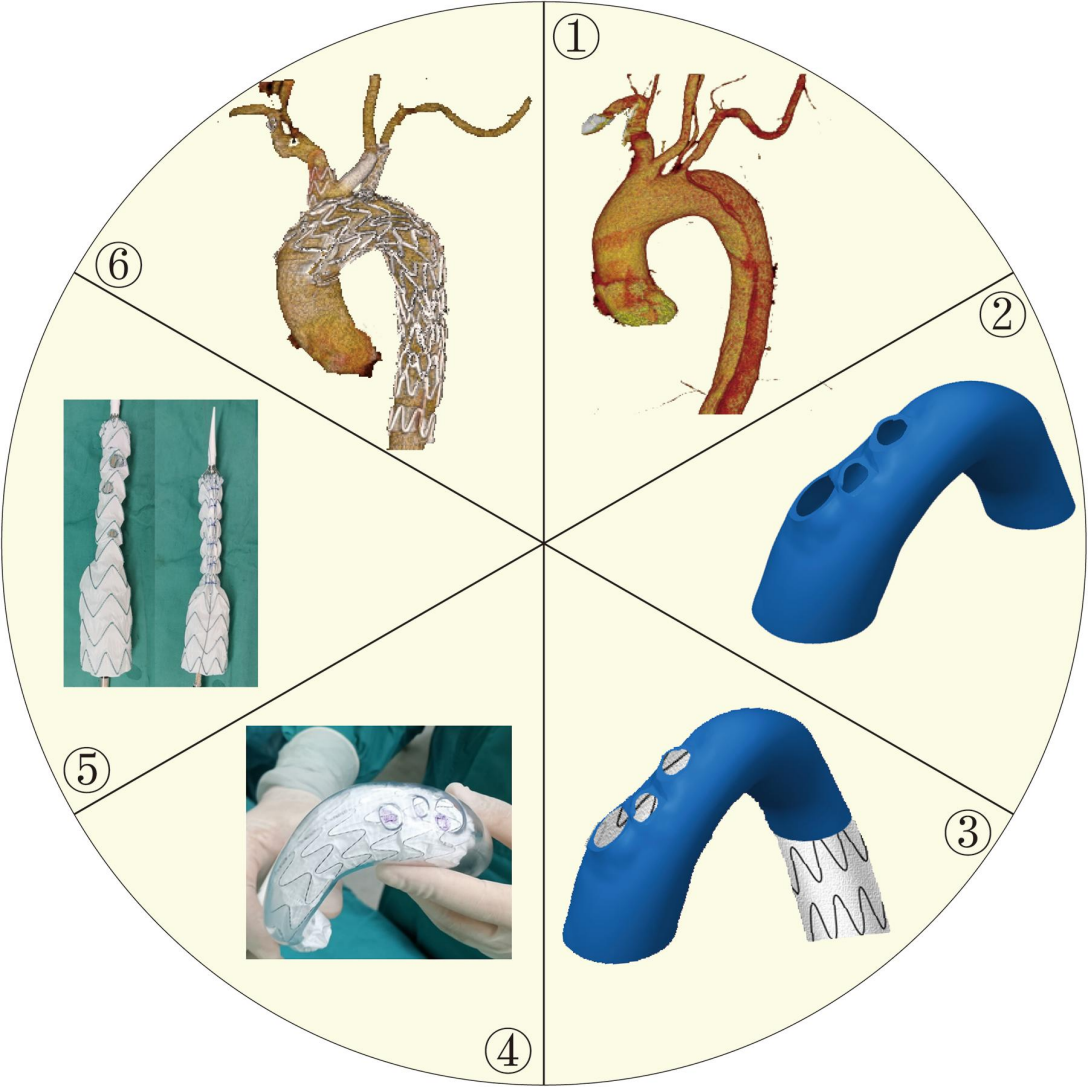
Standardized 3D-printing guided procedure. ① Preoperative CTA (Computed Tomography Angiography) was conducted to gather baseline data.② Completion of 3D vascular model based on CTA. ③ Release a stent graft in a 3D vascular model. ④ Mark the position of fenestration and modify the stent graft. ⑤ Apply the diameter reduction technique. ⑥ Accomplish F/B-TEVAR.

- Preoperative Computed Tomography Angiography (CTA) was conducted to gather baseline data;
- Intraoperatively, PMSG preparation was facilitated using conventional measurements or 3D printing-assisted technology. Simple fenestration or suturing of internal/external branch stents was performed at the branch artery orifices. Subsequently, the main stent graft underwent a temporary diameter reduction using sutures;
- The entire endovascular repair procedure was then executed. (Figure 1)

### 2.5 Operation steps

#### 2.5.1 Preoperative data collection

Preoperative 1 mm slices contrast enhanced CT angiography (ceCTa) scanning of the aorta was utilized to assess and measure the lesion, including the type (dissection, aneurysm, aortic arch ulcer), lesion length, diameters of the proximal and distal landing zones, and branch diameters. Once suitability for F/B-TEVAR was confirmed based on the patient's lesion and anatomy, surgical treatment commenced.

#### 2.5.2 Graft selection

Aortic stents with a posterior release structure and proximal bare stent structure were selected for intraoperative reconstruction, allowing for a larger scaffold gap to facilitate fenestration position placement. The utilized aortic stents include Ankura (Lifetech Scientific Company, China), Captiva (Medtronic Company, USA), Hercules (Microinvasive Company, China), Youyan (China), and Zenith (Cook Company, USA).

Stents for branched arteries primarily consist of covered options such as Fluency (Bard Company, USA), Viabahn (Gore, USA), and Silverflow (Lifetech Scientific, China). Other options include Lifestream (Bard Company, USA), VBX (Gore Company, USA), as well as spherical expansion bare stents and self-expanding bare stents including Omnilink and Absolute Pro (Abbott Company, USA), Luminex (Bard Company, USA), and Smart (Cordis Company, USA).

#### 2.5.3 Stent graft preparation

**Figure 2:**
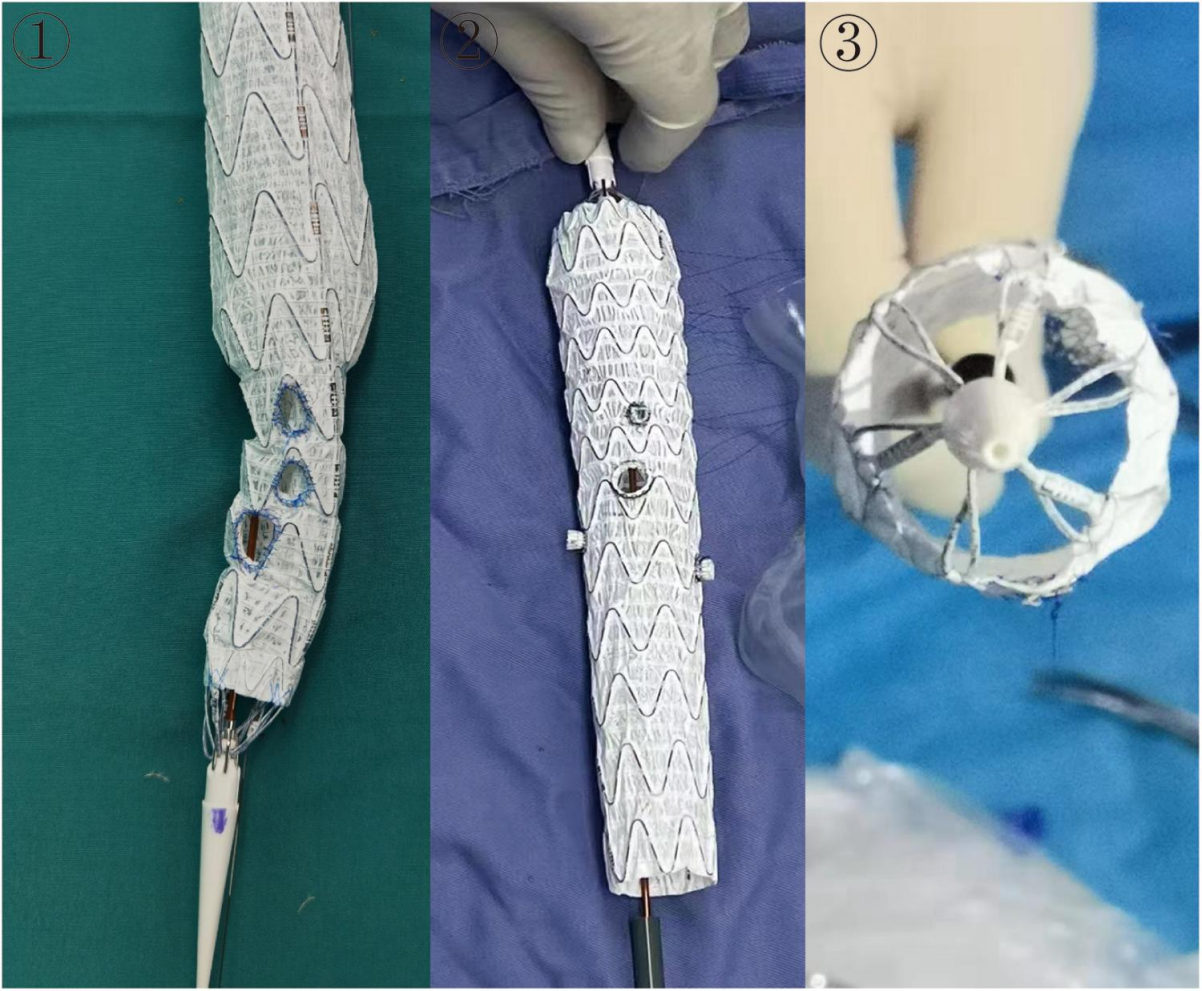
Stent graft preparation. ① Simple fenestration. ② External branching. ③ Internal branching.

A suitable thoracic aortic stent graft with a posterior release system was selected for real-time PMSG based on measurements from 3Mensio or Endosize software, utilizing either conventional measurement methods or assisted by 3D printing-assisted technology for fenestration positioning. Fenestration holes were created using an electrocautery pen based on the localization of the patient's branch arteries on the aortic arch. Following creation, the fenestration underwent treatment using simple fenestration, external branching, or internal branching methods. (Figure 2) Typically, internal branching is employed for fenestration at risk of internal leakage, while simple fenestration suffices for lesions not involving these risks. Additionally, appropriate guidewire catheters, arterial sheaths, and dilation balloons were prepared [For detailed methods, refer to (2)].

### 2.6 Surgical operations

In most cases, access for the aortic stent graft was obtained via one side of the femoral artery, while access for branch artery stent grafts utilized the left brachial artery, left common carotid artery, and right axillary artery, each exposed separately. The PMSG was delivered and partially released, and branch arteries were sequentially selected through the fenestrations. Local angiography confirmed branch artery selection, followed by wire exchange and gradual branch stent deployment. The bridging site was routinely dilated using a dilatation balloon. Finally, aortic stent implantation proceeded distally or proximally to the PMSG site, depending on the lesion, ensuring complete repair of the thoracic aortic lesion. Postoperative follow-up included instruction for CTA.

### 2.7 Postoperative management and monitoring

Depending on intraoperative bleeding, anesthesia duration, patient's baseline health, and other factors, patients are transferred to the intensive care unit (ICU) for close monitoring of vital signs. Monitoring focuses on cardiovascular and cerebrovascular conditions, checking for ischemic cerebral infarction, myocardial infarction, vascular access site complications such as bleeding and pseudoaneurysm formation, and assessing for diffuse coagulation dysfunction (DIC) or organ ischemic failure in cases of significant intraoperative hemorrhage. Additionally, attention is given to patients with poor baseline conditions for signs of in-stent infections, poor wound healing, and other postoperative complications.

### 2.8 Follow-up

All patients underwent regular follow-up at the outpatient clinic at 3 months, 6 months, and 1-year post-procedure, followed by annual check-ups. Continuous monitoring included CTA scans to assess lesion repair, detect endoleak, evaluate stent migration, and confirm branch artery patency. General patient condition was assessed, with special attention to mortality and any deaths related to the F/B-TEVAR procedure. The follow-up endpoint was defined as patient death or loss to follow-up.

### 2.9 Data collection

#### 2.9.1 General information

Baseline data, including age, gender, past medical history, and smoking/alcohol use, were collected via patient interviews and chart review.

#### 2.9.2 Imaging data

Pre and postoperative CTA images were reviewed to collect the primary diagnosis, evaluate branch artery involvement, and assess for the presence of aortic wall pathology. The focus of this phase was the systematic evaluation of aortic wall pathology, encompassing aortic atherosclerotic plaque, focal arch dilation, mural thrombus, and minute intramural hematomas. Two independent researchers assessed the images to identify the following non-major findings: small aortic plaques (maximum diameter <5 mm), focal dilatation of the aortic arch (>50% increase relative to the adjacent normal luminal diameter), minute intramural hematomas (thickness <5 mm), and mural thrombus. In the event of a disagreement, a third researcher with extensive clinical experience was invited to achieve consensus.

#### 2.9.3 Procedural details and disease outcomes

Including the surgical approach (assisted by 3D printing-assisted technology or not), operative duration, hemorrhage, number of supra-aortic branch reconstructions, and short-term postoperative outcomes.

#### 2.9.4 Follow-up data

Follow-up duration was defined as the time from the discharge date to the most recent CTA examination. A follow-up period exceeding five years (i.e., >1,826 days) was considered completed. Patient survival status was determined through telephone interviews and classified as routine follow-up, completed follow-up, survival with complications, death, or loss to follow-up.

### 2.10 Data analysis

Statistical analysis was performed using SPSS (version 26.0). Continuous variables conforming to a normal distribution were analyzed using one-way analysis of variance (ANOVA) or t-tests or Mann–Whitney U test and were presented as mean standard deviation ($\bar{x}\pm s$). Categorical variables were compared using the chi-square test. A significance level of *α*=0.05 was established, with *P* < 0.05 considered statistically significant.

To identify independent risk factors, a two-step multivariable logistic regression analysis was conducted. Initially, all candidate variables were assessed using univariable logistic regression; those with a P-value < 0.05 were selected for inclusion in the subsequent multivariable model. The results of the multivariable analysis are presented as odds ratios (ORs) with corresponding 95% confidence intervals (CIs). All enrolled cases who under the procedure were included in the final analysis, regardless of intraoperative outcomes or postoperative survival status, to avoid selection bias. Model fit was evaluated using the −2-log likelihood(-2LL), Cox & Snell R (2). Calibration was evaluated with the Hosmer-Lemeshow goodness-of-fit test, and discriminative ability was quantified by the area under the receiver operating characteristic curve (AUC).

## 3 Results

### 3.1 General results

**Figure 3:**
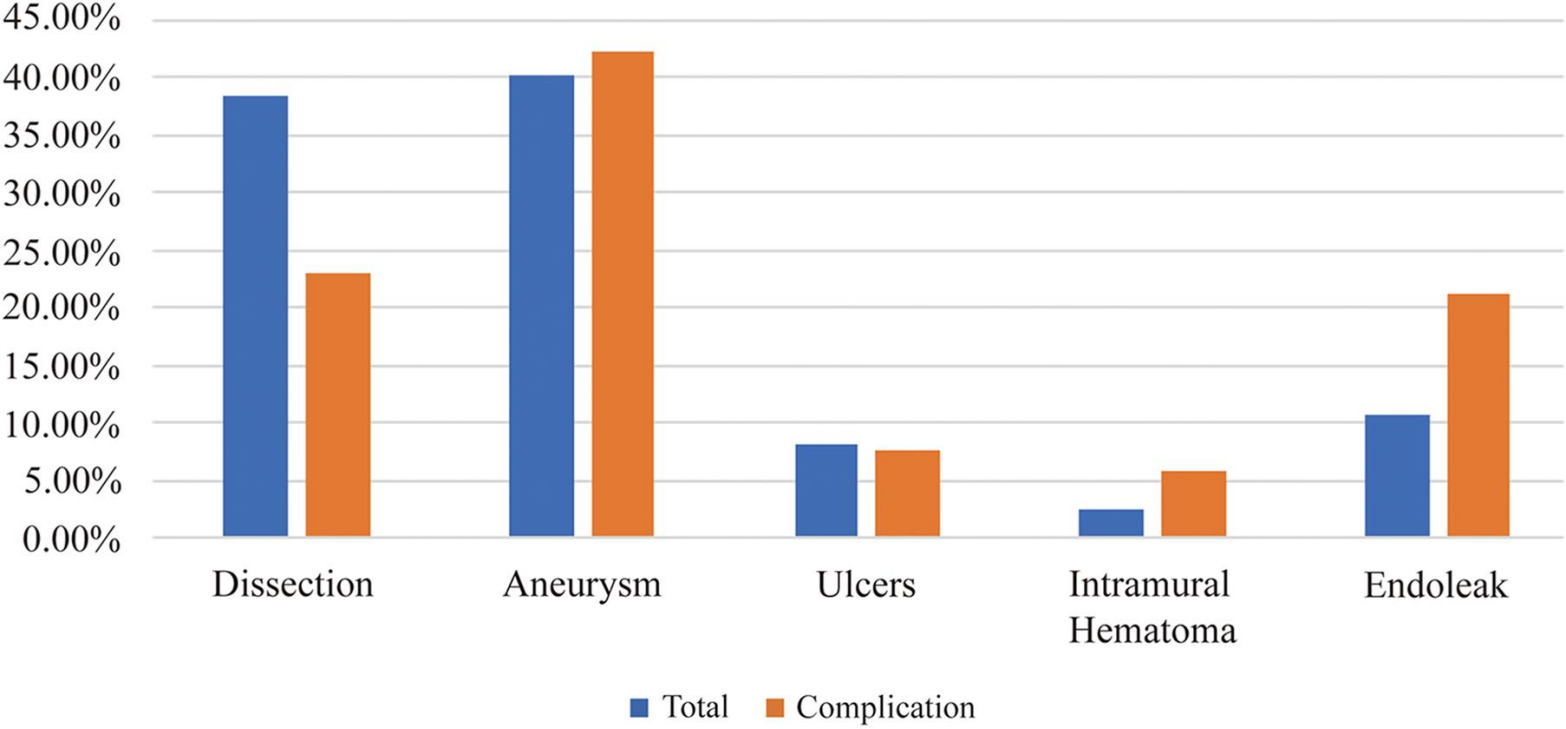
Percentage of primary diseases.

From February 2018 to December 2024, 246 patients underwent F/B-TEVAR for thoracic aortic lesions with multibranch reconstruction at 4 medical centers in China. The median follow-up duration was 828 days. The 1-year and 5-year follow-up completion rates were 72.8% and 9.8%, respectively, with a loss-to-follow-up rate of 8.1%. Of these, 211 were male and 35 were female, aged between 30 and 86 years (mean age: 62.5 ± 12.1 years). Diagnoses included 100 patients with aortic aneurysm, 94 with aortic dissection, 20 with thoracic aortic ulcer, 26 with previous postoperative endoleak of aortic lesions, and 6 with intermural hematoma of the thoracic aorta. All branches were successfully reconstructed in one stage without intermediate open surgery, except in cases of intraoperative retrograde tears and intraoperative deaths. Among these patients, 171 cases (69.5%) were treated with 3D printing-assisted F/B-TEVAR. Significant differences were observed in the distribution of pathological types among the groups (*χ*² = 13.945, *p* = 0.007; Fisher's exact test, *p* = 0.007). As shown in the bar chart, preoperative endoleak was markedly more frequent in the complication group (20.8%, 11/53) than in the non-complication group (7.8%, 15/193), while the incidence of aortic dissection was significantly lower in the complication group (22.6%, 12/53) compared to the non-complication group (22.6%, 12/53 vs 42.5%, 82/193). For the aneurysm group, the incidence rates were similar between the two groups (Figure 3). Specifically clinical data are presented in Table 1.

**Table 1:** Clinical data and risk factors of 246 patients with F/B-TEVAR.

Variable	Total cases (*n* = 246)	Complication cases (*n* = 53)	Non-complication cases(*n* = 193)
Age	62.52 ± 12.11	61.98 ± 11.8	62.63 ± 11.917
Gender			
Male	211 (85.8%)	42 (79.2%)	169 (87.6%)
Female	35 (14.2%)	11 (20.8%)	24 (12.4%)
The type of pathology			
Aortic Dissection	94 (38.2%)	12 (22.6%)	82 (42.5%)
Aortic Aneurysm	100 (40.7%)	23 (43.4%)	77 (39.9%)
Preoperative Endoleak	26 (10.6%)	11 (20.8%)	15 (7.8%)
Aortic Ulcer	20 (8.1%)	4 (7.5%)	16 (8.3%)
Aortic Intermural Hematoma	6 (2.4%)	3 (5.7%)	3 (1.6%)
Aortic wall pathology			
Lesions Involving Branch Arteries	17 (6.9%)	13 (24.5%)	4 (2.1%)
Aortic Atherosclerotic Plaque	64 (26.0%)	34 (64.2%)	30 (15.5%)
Focal arch dilatation	46 (18.7%)	24 (45.3%)	22 (11.4%)
mural thrombus	49 (20.0%)	28 (52.8%)	21 (10.9%)
Intermural Hematoma	40 (16.3%)	20 (37.7%)	20 (10.4%)
Chronic Disease			
History of Aortic Surgery	61 (24.8%)	16 (30.2%)	45 (23.3%)
Hypertension	109 (44.3%)	38 (71.7%)	71 (36.8%)
Diabetes	22 (8.9%)	6 (11.3%)	16 (8.3%)
Coronary Artery Disease	25 (10.2%)	8 (15.1%)	17 (8.8%)
Hyperlipidemia	13 (5.3%)	3 (5.7%)	10 (5.2%)
Stroke	16 (6.5%)	14 (26.4%)	2 (1.0%)
Smoking	45 (18.3%)	30 (56.6%)	15 (7.8%)
Alcohol Consumption	17 (6.9%)	8 (15.1%)	9 (4.7%)
Procedural Details			
3D printing-assisted technology	171 (69.5%)	29 (54.7%)	142 (73.6%)
Triple-branched reconstruction	144 (58.5%)	32 (60.4%)	112 (58.0%)
Two-branched reconstruction	102 (41.5%)	21 (36.6%)	81 (42.0%)
Follow up			
1-year follow up completed	179 (72.8%)	35 (66.0%)	144 (74.6%)
5-year follow up completed	24 (9.8%)	7 (13.2%)	17 (8.8%)
Loss-of follow up	20 (8.1%)	4 (7.5%)	16 (8.3%)
Median	828 [day]	801 [day]	745 [day]

### 3.2 Complication results

There were 53 cases of postoperative complications, resulting in an overall complication rate of 21.5%. Detailed results are as follows.

- Endoleak. Intraoperative completion digital subtraction angiography (DSA) was routinely performed immediately following aortic endovascular repair. Postoperatively, CTA serves as the primary imaging modality for surveillance. An endoleak was defined as imaging-confirmed persistent blood flow within the native aortic aneurysm sac but outside the graft lumen. Endoleaks were detected in 21 patients, comprising 14 type Ⅰ and 7 type Ⅱ cases, no type Ⅲ、Ⅳ or Ⅴ endoleaks were observed. Among these, 3 endoleaks were identified intraoperatively on completion DSA, while the remaining 18 were discovered during follow-up surveillance via CTA.
- Ischemic stroke. Ischemic stroke was the second most common postoperative complication in this study, occurring in 13 patients, all within the perioperative period. All patients presented with postoperative changes in muscle strength and consciousness as the primary manifestations, and stroke was subsequently confirmed by CT scans. Among these, three cases resulted in perioperative death.
- Retrograde type A dissection. Retrograde type A aortic dissection (RTAD) was the most severe postoperative complication in this study, occurring in 6 patients. Among these, 3 resulted in intraoperative death; 2 underwent immediate conversion to open surgery intraoperatively and survived; and 1 presented with chest pain 12 hours postoperatively, was diagnosed with RTAD by CTA, and survived after emergency open surgery.

Among the remaining cases, there were 2 of spinal cord ischemia, 3 of approach complications, 2 of incision complications, 2 of renal insufficiency, 1 of postoperative DIC, 1 of cardiogenic shock, and 2 of severe infection. 8 cases of complication-related death were recorded: 3 from retrograde tear of the aorta, 3 from ischemic stroke, 1 from cardiogenic sudden death, and 1 from severe infection. The complications are summarized in detail in Table 2.

**Table 2:** Postoperative complications in 246 cases of F/B-TEVAR.

Postoperative complications	Statistical values
Endoleak	21 (8.54%)
Ischemic Stroke	13 (5.28%)
Spinal Cord Ischemia	2 (0.81%)
Retrograde Type A Dissection	6 (2.44%)
Approach Complications	3 (1.22%)
Incision Complications	2 (0.81%)
Renal Insufficiency	2 (0.81%)
Disseminated Intravascular Coagulation	1 (0.41%)
Cardiogenic Shock	1 (0.41%)
Severe Infection	2 (0.81%)

### 3.3 Risk factors analysis

Univariate logistic analysis found no statistically significant associations between postoperative complications and variables such as age, gender, number of branch artery reconstructions, history of alcohol consumption, hypertension, coronary heart disease, diabetes, hyperlipidemia, or history of aortic surgery (all *P* > 0.05). In contrast, the application of 3D printing-assisted technology, a history of previous cerebral infarction, smoking, lesions involving branch arteries, aortic atherosclerotic plaques, mural thrombus, minute intermural hematoma, and focal arch dilatation were significantly associated with the development of postoperative complications (all *P* < 0.05).

Incorporating the above statistically significant risk factors into a multivariable logistic regression analysis, we found that a history of previous cerebral infarction [OR 12.050; 95% CI (1.804–80.496); *P* = 0.01], a history of smoking [OR 4.759; 95% CI (1.611–14.060); *P* < 0.01], lesions involving branch arteries [OR 8.176; 95% CI (1.689–39.571); *P* < 0.01], aortic atherosclerotic plaques [OR 3.003; 95% CI (1.040–8.667); *P* = 0.042], mural thrombus [OR 3.388; 95% CI (1.071–10.721); *P* = 0.038], and minute intermural hematomas [OR 4.122; 95% CI (1.230–13.810); *P* = 0.022] were independent risk factors for the development of postoperative complications in F/B-TEVAR surgery.

Notably, among the surgical factors, the application of 3D printing-assisted technology [OR 0.434; 95% CI (0.232–0.814); *P* = 0.009] was identified as a protective factor in univariable analysis, but it did not demonstrate an independent effect after multivariable adjustment.

The multivariable model demonstrated good fit, with a −2 log likelihood (−2LL) value of 94.428, Cox & Snell R² of 0.435, and Nagelkerke R² of 0.604. The Hosmer–Lemeshow goodness-of-fit test yielded a P value of 0.079, indicating adequate calibration. The area under the receiver operating characteristic curve (AUC) was 0.902, suggesting excellent discriminative ability of the model. (See Table 3 for details.)

**Table 3:** Risk factors analysis.

Variable	Univariable Logistic Regression		Multivariable Logistic Regression Analysis			
	*P* value	OR	*β*	*P* value	OR	95%CI
Gender	0.129	1.844				
Age	0.789	0.997				
3D printing-assisted technology	*p* < 0.01*	0.434	−0.88	0.872	0.915	0.313–2.675
Number of branch artery reconstructions	0.759	1.102				
Hypertension	0.427	1.470				
Coronary Artery Disease	0.804	0.837				
Diabetes	0.511	0.712				
Hyperlipidemia	0.414	0.571				
Stroke History	*p* < 0.01*	19.469	2.49	0.010*	12.050	1.804–80.496
History of Aortic Surgery	0.306	1.422				
Smoking	*p* < 0.01*	10.000	1.56	0.005*	4.759	1.611–14.060
Alcohol Consumption	0.205	1.946				
Lesions Involving Branch Arteries	*p* < 0.01*	8.734	2.10	0.009*	8.176	1.689–39.571
Aortic Atherosclerotic Plaque	*p* < 0.01*	6.182	1.10	0.042*	3.003	1.040–8.667
Intermural Hematoma	*p* < 0.01*	2.800	1.42	0.022*	4.122	1.230–13.810
mural thrombus	*p* < 0.01*	5.412	1.22	0.038*	3.388	1.071–10.721
Focal arch dilatation	0.010*	3.481	0.83	0.126	2.295	0.792–6.654

*Variables with *P* > 0.05 in univariable analysis were not entered into the multivariable model. Model fit: −2 log likelihood (−2LL) = 94.428; Cox & Snell R² = 0.435; Nagelkerke R² = 0.604; Hosmer–Lemeshow test: *χ*²=14.090, *P* = 0.079; Area under the ROC curve (AUC) = 0.902 (95%CI: 0.841–0.962, *p* < 0.001). *.

## 4 Discussion

**Figure 4:**
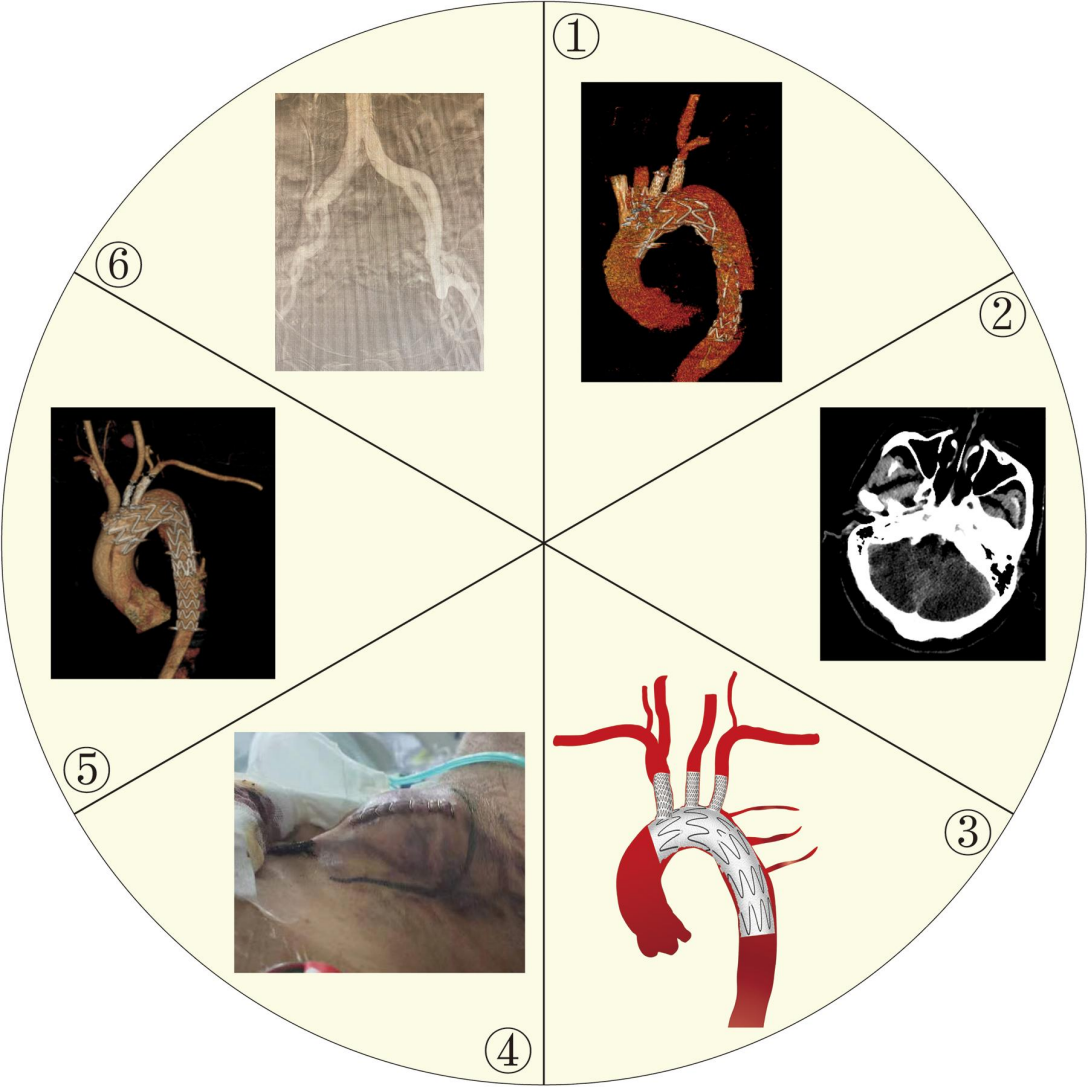
Common post-operative complications. ① A case of postoperative endoleak. ② A case of postoperative large cerebellar hemisphere infarction. ③ Schematic of postoperative spinal cord ischemia. ④ A case of incisional hematoma. ⑤ A case of retrograde aortic dissection. ⑥ A case of postoperative lower extremity arterial occlusion.

The use of F/B-TEVAR for treating complex aortic arch disease has increased in recent years, showing advantages over open aneurysm repair, including higher perioperative survival rates. A systematic evaluation of 1,532 patients indicated that endovascular repair had a lower 30-day mortality rate (1.6%) compared to open surgery (4.8%) (3). The survival benefit was even more significant in high-risk patients, with a 30-day mortality rate of 4.7% versus 19.2% for open repair (4). F/B-TEVAR also eliminates the need to cross-clamp or open the aorta, resulting in less blood loss and shorter recovery periods, and decreasing aneurysm-related mortality (5). However, it comes with a high incidence of postoperative complications, including systemic and local complications related to the endograft device. Approximately 19%-24% of endovascular thoracic aortic aneurysm repairs require secondary re-intervention, and of these, as many as 38% of patients require secondary intervention, with a late complication rate of up to 41% (6–9). Common complications are listed below and in Figure 4.

### 4.1 Postoperative endoleak

Particularly type I endoleak, was the most common complication in our study. Hogendoorn W and Schlösser FJ et al. reported postoperative endoleak in 18% of patients using the chimney graft technique for thoracic aortic repair, with type ⅠA being the most frequent at 6.4% (10). The estimated prevalence of endoleak in all indications for TEVAR is 3.9-30.5% (5, 11, 12). Endoleaks are closely related to the anatomical characteristics of the thoracic aorta, which requires thicker overlay stents and longer anchoring and sealing zones due to its larger diameter and variable arch anatomy. In our study, 3D printing-assisted F/B-TEVAR effectively reduced complications as evidenced by regression analyses (*p* = 0.009), particularly postoperative endoleak, by allowing individualized and precise positioning of branch arterial openings on the aortic arch. Most endoleaks occurred in preexisting patients were attributed to minima aortic stent oversizing (0-5%). In later clinical practice, we used 10% oversize for aortic coarctation and 20% for aortic aneurysm, resulting in almost no type IA endoleak.

### 4.2 Cerebrovascular events

Embolic stroke incidence after TEVAR is reported to be 4%-8%, comparable to open surgery (13). Factors such as mobile atherosclerosis in the aortic arch, a history of previous stroke, and proximal graft deployment increase the risk (14). Vallabhajosyula P, Szeto W Y, Desai N, Komlo C, Bavaria J E. in their study report that the incidence of perioperative stroke in TEVAR may be 4% to 8% (15–17), which is comparable to our study's finding of an incidence of 5.33% for postoperative stroke. Prolonged operative time, catheter or guidewire manipulation, tortuous vertebral arch anatomy, and poor perioperative blood pressure control were associated with ischemic stroke. The pre-opening technique with diameter reduction (40-50%) during PMSG operation maintained adequate brain blood supply, reducing cerebral complications (2). Cerebrovascular accidents occurred in patients with large arch aneurysm lumens, extensive thrombus, and severe aortic calcification, often due to intraoperative manipulation dislodging thrombus or plaque. Aortic coarctation patients with minimal atherosclerosis had a low incidence of such events. Multifactorial logistic regression indicated that vascular atherosclerotic plaque formation, intramural thrombosis, and stroke history are independent risk factors for perioperative ischemic stroke. Therefore, perioperative screening for stroke history is crucial, and blood pressure management with antiplatelet drugs, lipid-lowering drugs, or beta-blockers is recommended to reduce complications. Strict adherence to operative procedures and gentle handling during surgery are essential. Using a brain umbrella for high-risk patients has effectively reduced stroke incidence in our practice.

### 4.3 Spinal cord ischemia

A complication of F/B-TEVAR, occurs due to stent-grafts covering spinal trophoblastic arteries (4). It may occur in 3%-5.6% of patients (6), with permanent ischemia in 2%. Our study reported a 0.8% incidence of paraplegia, which is much lower than that reported in the relevant literature. Preoperative lumbar cistern drainage can prevent paraplegia (18, 19) but may increase puncture-related complications. We minimized spinal cord artery coverage and avoided using two long body stents simultaneously. In patients with long lesion segments, we performed staged surgeries to avoid extensive ischemia. Perioperative measures included increasing mean arterial pressure, maintaining hematocrit levels, and adequate anticoagulation and vasodilation.

### 4.4 Retrograde aortic tears

Performing endovascular stent-graft implantation in patients with aortic coarctation risks access coarctation tears and downstream lesion progression. The incidence of retrograde aortic tears is reported to be 1.33%-3%, with a mortality rate of up to 42% (20). In our study, 2.44% of patients experienced postoperative retrograde tears, consistent with existing research. All patients with retrograde tears had intramural hematomas in the proximal loading zone (PLZ). We avoided proximal balloon molding. Although retrograde entrapment is not a direct complication of F/B-TEVAR, this adjunctive technique allows for lower threshold coverage of the proximal arch, enabling emergency treatment of complex arch disorders. In our study, 2 of 6 retrograde entrapment cases involved a primary aortic arch hematoma, and 4 involved aortic dissection. Retrograde aortic dissection risk was higher with double left subclavian artery (LSA) + left carotid artery (LCA) reconstruction (4/102, 3.92%) compared to triple innominate artery (INA) + LSA + LCA reconstruction (2/144, 1.39%). This is related to the specific anatomy of the aortic arch.

In two-branch reconstruction, the covered segment was at the anterior edge of the LCA, and the bare stent was at the anterior edge of the IA, stressing the great curvature and potentially causing the bare stent to pierce the aortic intima, leading to retrograde aortic dissection (RTAD). Three-branch reconstruction placed the bare stent in the ascending aorta, reducing RTAD risk. Only two cases of RTAD occurred in our group with three-branch reconstruction; one was due to operator inexperience, and the other was related to vessel development and strong stent support. For two-branch reconstruction needing a proximal LCA loading zone, we used a single-branch stent with a single open fenestration, eliminating RTAD due to the absence of a proximal bare stent.

### 4.5 Access complications

Ischemic issues post-TEVAR, occurred in 9% of cases, higher than after open surgical repair (21). In our study, 3 of 246 cases (1.22%) had postoperative occlusion of the iliac and lower limb arteries due to access injuries.

### 4.6 Coagulation disorders

Coagulation dysfunction is common in thoracic aortic aneurysms (TAA), often due to depletion of coagulation factors by thrombosis after endovascular repair. Patients’ coagulation function should be monitored within 24 hours post-operation, and medication adjusted based on laboratory results to prevent complications.

### 4.7 Incisional complications

This study reported one case of postoperative cervical hematoma and one case of postoperative axillary hematoma. Careful intraoperative suturing of the access artery is essential, especially in the groin, to prevent hemostasis issues and lymphatic leakage. Additionally, F/B-TEVAR may cause complications such as post-stenting syndrome, incision infection, and upper limb ischemia, which are not discussed here due to space limitations.

### 4.8 3D printing-assisted technology

3D printing-assisted technology emerged as a potential protective factor, showing statistical significance in univariate regression analysis [OR 0.434; 95% CI (0.232–0.814); *P* = 0.009], which aligns with our clinical observations. Its diminished effect in the multivariate model is likely attributable to the timeline of its adoption and associated learning curve. Since its introduction at one of our four medical centers in 2018, the technology required a learning curve. It was not until 2022, following key breakthroughs in 3D modeling at our research group, that we began to widely share this 3D printing-assisted technology with other medical centers. Clinically, the maturation of 3D printing-assisted technology has been observed to significantly reduce the number of catheterizations and guidewire exchanges required for target vessel cannulation. This technical advantage directly translates to reduced mechanical trauma, critical factor given recent evidence linking the frequency of endovascular manipulations to complications such as retrograde type A dissection (RTAD) and stroke (22). Moreover, more precise positioning reduces contrast medium volume, thereby mitigating nephrotoxicity and the cytotoxic effect of contrast media on the vascular endothelium (23), a benefit particularly relevant in patients with pre-existing endothelial dysfunction.

### 4.9 Risk factors

A closer examination of the independent risk factors identified in this study reveals a unified pathophysiological mechanism that underpins both the clinical histories and aortic wall pathologies: active arterial wall inflammation, endothelial dysfunction, and accelerated atherosclerosis. The formation of mural thrombus and intramural hematoma has been associated with impaired endothelial function (24). Smoking contributes to vascular damage through a dual mechanism: it not only accelerates atherosclerosis but also increases vessel wall fragility, potentially by promoting extracellular matrix degradation, thereby rendering the aorta more susceptible to injury (25). Moreover, involvement of the supra-aortic branches reflects advanced anatomical deterioration, while a history of stroke indicates that the patient's systemic atherosclerotic burden has reached a threshold suggestive of diffuse and irreversible vascular disease. To some extent, factors that promote vascular injury—such as local arterial wall pathology and local anatomical hemodynamic alterations—persist throughout the patient's lifetime, creating an ongoing risk of late clinical deterioration (26). Consequently, even with optimal intraoperative anatomical reconstruction, patients with a prior stroke face a higher risk of postoperative vascular disease progression.

The distribution of pathological types significantly influences the occurrence of complications. As shown in Figure 3, preoperative endoleak was significantly more frequent in the complication group (20.8%, 11/53) than in the non-complication group (7.8%, 15/193), which may be attributed to the higher proportion of aortic aneurysm patients with advanced age, severe atherosclerosis, tortuous and stenotic vessels, and poor branch artery conditions—factors that predispose to endoleak, cerebral infarction, and mortality. In contrast, aortic dissection was significantly less common in the complication group (22.6%, 12/53) compared to the non-complication group (42.5%, 82/193). Although patients with aortic dissection are generally younger and tend to have more normal aortic morphology, less tortuosity and stenosis, and healthier branch arteries—which may reduce the risk of complications such as endoleak and stroke—retrograde type A aortic dissection (RTAD) remains a concern due to the underlying pathophysiological characteristics of dissected aortas and potential abnormalities in arterial development. For aortic aneurysm, the incidence rates were similar between the two groups, suggesting that other factors may play a more prominent role in determining complication risk in this subgroup. Medium- and long-term effects require further follow-up.

### 4.10 Study limitations

It must be acknowledged that, as a retrospective study, this research inherently possesses selection bias and information bias. The accuracy of the findings is contingent upon the completeness and veracity of medical records, and there exist confounding factors that cannot be entirely excluded, such as variations in surgeon experience and technical skillset, as well as differences in selection of the landing zone. In the management of this multicenter study, efforts were made to standardize surgical approaches through technical exchanges and the uniform utilization of single-source 3D vascular models. Furthermore, all data collection was performed by the same group of investigators to mitigate multicenter heterogeneity as much as possible. Despite these efforts, the risk of such confounding could not be completely eliminated.

Additionally, in the multivariate analysis, the occurrence of only 53 complication events limited the number of reliable variables that could be included in the model, as some variables did not meet the requirement of ≥10 events per variable. This may affect the stability of the model and increase the risk of overfitting. Finally, this study identifies associations rather than causality; therefore, its clinical generalization warrants further validation through additional research.

## 5 Conclusion

Our retrospective study identified aortic anatomical factors such as aortic atherosclerotic plaque, minute intermural hematomas, mural thrombus, history of previous cerebral infarction, history of smoking, and lesions involving branch arteries as independent risk factors for postoperative complications after F/B-TEVAR for complex aortic arch lesions. The use of 3D printing-assisted technology can reduce postoperative complications by precisely locating branch openings, aiding clinicians in identifying high-risk patients and selecting optimal surgical approaches and perioperative interventions.

## Data Availability

The original contributions presented in the study are included in the article/Supplementary Material, further inquiries can be directed to the corresponding author.

## References

[1] Bavaria JE Appoo JJ Makaroun MS Endovascular stent grafting versus open surgical repair of descending thoracic aortic aneurysms in low-risk patients: a multicenter comparative trial. J Thorac Cardiovasc Surg. (2007) 133(2):369–377. 10.1016/j.jtcvs.2006.07.040 17258566

[2] Liu Z Tong Y Yu T Treatment of thoraco-abdominal aortic disease with fenestrated stent-graft or branch stent-graft technique guided by 3D printing. Chin J Gen Surg. (2019) 34(3):213–216. 10.3760/CMA.J.ISSN.1007-631X.2019.03.006

[3] Lederle FA Kane RL MacDonald R Wilt TJ. Systematic review: repair of unruptured abdominal aortic aneurysm. Ann Intern Med. (2007) 146(10):735–741. 10.7326/0003-4819-146-10-200705150-00007 17502634

[4] Davies RR Gallo A Coady MA Novel measurement of relative aortic size predicts rupture of thoracic aortic aneurysms. Ann Thorac Surg. (2006) 81(1):169–177. 10.1016/j.athoracsur.2005.06.026 16368358

[5] Makaroun MS Dillavou ED Wheatley GH Cambria RP, Gore TAGI. Five-year results of endovascular treatment with the gore TAG device compared with open repair of thoracic aortic aneurysms. J Vasc Surg. (2008) 47(5):912–918. 10.1016/j.jvs.2007.12.006 18353605

[6] Matsumura JS Cambria RP Dake MD International controlled clinical trial of thoracic endovascular aneurysm repair with the zenith TX2 endovascular graft: 1-year results. J Vasc Surg. (2008) 47(2):247–257. discussion 257. 10.1016/j.jvs.2007.10.032 18241743

[7] Scali ST Beck AW Butler K Pathology-specific secondary aortic interventions after thoracic endovascular aortic repair. J Vasc Surg. (2014) 59(3):599–607. 10.1016/j.jvs.2013.09.050 24571937 PMC4120941

[8] Lederle FA Freischlag JA Kyriakides TC Long-term comparison of endovascular and open repair of abdominal aortic aneurysm. N Engl J Med. (2012) 367(21):1988–1997. 10.1056/NEJMoa1207481 23171095

[9] participants Et. Endovascular aneurysm repair versus open repair in patients with abdominal aortic aneurysm (EVAR trial 1): randomised controlled trial. Lancet. (2005) 365(9478):2179–2186. 10.1016/S0140-6736(05)66627-5 15978925

[10] Hogendoorn W Schlosser FJ Moll FL Sumpio BE Muhs BE. Thoracic endovascular aortic repair with the chimney graft technique. J Vasc Surg. (2013) 58(2):502–511. 10.1016/j.jvs.2013.03.043 23697513

[11] Dake MD Miller DC Mitchell RS Semba CP Moore KA Sakai T. The “first generation” of endovascular stent-grafts for patients with aneurysms of the descending thoracic aorta. J Thorac Cardiovasc Surg. (1998) 116(5):689–703. discussion 703-684. 10.1016/S0022-5223(98)00455-3 9806376

[12] Lal BK Zhou W Li Z Predictors and outcomes of endoleaks in the veterans affairs open versus endovascular repair (OVER) trial of abdominal aortic aneurysms. J Vasc Surg. (2015) 62(6):1394–1404. 10.1016/j.jvs.2015.02.003 26598115

[13] Xie E Yang F Liu Y Timing and outcome of endovascular repair for uncomplicated type B aortic dissection. Eur J Vasc Endovasc Surg. (2021) 61(5):788–797. 10.1016/j.ejvs.2021.02.026 33846073

[14] Zhong J Osman A Tingerides C Technique-Based evaluation of clinical outcomes and aortic remodelling following TEVAR in acute and subacute type B aortic dissection. Cardiovasc Intervent Radiol. (2021) 44(4):537–547. 10.1007/s00270-020-02749-2 33388868

[15] Teufelsbauer H Prusa AM Wolff K Endovascular stent grafting versus open surgical operation in patients with infrarenal aortic aneurysms: a propensity score-adjusted analysis. Circulation. (2002) 106(7):782–787. 10.1161/01.CIR.0000028603.73287.7D 12176947

[16] Karthikesalingam A Holt PJ Hinchliffe RJ Nordon IM Loftus IM Thompson MM. Risk of reintervention after endovascular aortic aneurysm repair. Br J Surg. (2010) 97(5):657–663. 10.1002/bjs.6991 20235086

[17] Moulakakis KG Dalainas I Mylonas S Giannakopoulos TG Avgerinos ED Liapis CD. Conversion to open repair after endografting for abdominal aortic aneurysm: a review of causes, incidence, results, and surgical techniques of reconstruction. J Endovasc Ther. (2010) 17(6):694–702. 10.1583/1545-1550-17.6.694 21142475

[18] McDonald RJ McDonald JS Kallmes DF Intracranial gadolinium deposition after contrast-enhanced MR imaging. Radiology. (2015) 275(3):772–782. 10.1148/radiol.15150025 25742194

[19] Millen A Canavati R Harrison G Defining a role for contrast-enhanced ultrasound in endovascular aneurysm repair surveillance. J Vasc Surg. (2013) 58(1):18–23. 10.1016/j.jvs.2012.12.057 23490295

[20] Vallabhajosyula P Szeto WY Desai N Komlo C Bavaria JE. Type II arch hybrid debranching procedure. Ann Cardiothorac Surg. (2013) 2(3):378–386. 10.3978/j.issn.2225-319X.2013.05.08 23977611 PMC3741848

[21] Maldonado TS Rockman CB Riles E Ischemic complications after endovascular abdominal aortic aneurysm repair. J Vasc Surg. (2004) 40(4):703–709. discussion 709-710. 10.1016/j.jvs.2004.07.032 15472598

[22] Awiwi MO Kandemirli VB Kokash D Hossain F Gjoni M Odisio E Complications of thoracic endovascular aneurysm repair (TEVAR): a pictorial review. Curr Probl Diagn Radiol. (2024) 53(5):648–661. 10.1067/j.cpradiol.2024.05.018 38777715

[23] Magnusson MMM Gerk U Schüpbach G Rieger J Plendl J Marin I Microvascular changes following exposure to iodinated contrast media *in vitro*. A qualitative comparison to serum creatinine concentrations in post-cardiac catheterization patients. Microvasc Res. (2024) 153:104659. 10.1016/j.mvr.2024.104659 38286222

[24] Alibaz-Oner F Yurdakul S Aytekin S Direskeneli H. Impaired endothelial function in patients with takayasu’s arteritis. Acta Cardiol. (2014) 69(1):45–9. 10.1080/AC.69.1.3011344 24640521

[25] Ucar AK Ozdede A Kayadibi Y Adaletli I Melikoglu M Fresko I Increased arterial stiffness and accelerated atherosclerosis in takayasu arteritis. Semin Arthritis Rheum. (2023) 60:152199. 10.1016/j.semarthrit.2023.152199 37011578

[26] Bukun HO Uygunoglu U Senturk EF Duru G Kızılkılıç O Esatoglu SN Internal carotid artery involvement and stroke risk in takayasu arteritis: a case-control study. Rheumatol Int. (2026) 46(3):45. 10.1007/s00296-026-06075-7 41653289 PMC12882948

